# Hepatitis C virus seroprevalence among people who inject drugs and factors associated with infection in eight Russian cities

**DOI:** 10.1186/1471-2334-14-S6-S12

**Published:** 2014-09-19

**Authors:** Robert Heimer, Ksenia Eritsyan, Russell Barbour, Olga S Levina

**Affiliations:** 1 Department of Epidemiology of Microbial Diseases, Yale University School of Public Health, New Haven, CT, USA; 2 Center for Interdisciplinary Research on AIDS, Yale University, New Haven, CT, USA; 3 NGOStellit, St. Petersburg, Russian Federation

## Abstract

**Background:**

Behavioural surveillance among people who inject drugs (PWID) and testing for hepatitis C virus (HCV) and HIV is needed to understand the scope of both epidemics in at-risk populations and to suggest steps to improve their health.

**Methods:**

PWID were recruited using respondent-driven sampling (RDS) in eight Russian cities. A standardized survey was administered to collect sociodemographic and behavioral information. Blood specimens were obtained for serological testing for HCV and HIV-1. Data across the eight sites were pooled to identify individual-, network-, and city-level factors associated with positive HCV serostatus.

**Results:**

Among 2,596 PWID participating in the study, 1,837 tested positive for HCV (71%). The sample was 73% male and the mean age was 28. Very few PWID reported regular contact with harm reduction programs. Factors associated with testing positive for HCV were longer duration of injection drug use, testing positive for HIV-1, sharing non-syringe injection paraphernalia and water for rinsing syringes, and larger social network size. Factors negatively associated with HCV-positive serostatus were injecting with a used syringe and two city-level factors: longer mean RDS recruitment chain in a city and higher levels of injecting stimulants.

**Conclusions:**

HCV prevalence in all eight Russian cities is at the higher end of the range of HCV prevalence among PWID in Europe, which provides evidence that more resources, better prevention programs, and accelerated treatment targeting PWID are needed to control the HCV epidemic.

## Introduction

Hepatitis C virus (HCV) infection is a widespread and well-recognized consequence of injection drug use in many countries including Russia [1]. There are an estimated 1.8-2.2 million people who inject drugs (PWID) in Russia [2,3], but no nationwide data collection and surveillance system exists for HCV such as there is for HIV-1, so all data on HCV are local. The first HCV studies conducted more than a dozen years ago found that prevalence among PWID in Russia was 80% or higher [4,5]. These studies were conducted in Russian cities that also were experiencing a rapidly growing HIV epidemic among drug users, such as St. Petersburg and Togliatti [6-8]. Since then, few reports on the prevalence of HCV among PWID in different parts of the country have appeared in the peer-reviewed literature, but in those reports, prevalence has ranged from 54% in Barnaul to 95% in St. Petersburg, with intermediate levels in Moscow, Volgograd, and Irkutsk [9-11]. None of the study populations were tested more recently than 2006. Given this long interval, it is appropriate to revisit the issue of HCV prevalence among PWID in Russian cities. Such an inquiry is especially timely since the first direct-acting HCV antiviral medication, simeprevir, recently was approved by the Russian Ministry of Health [12].

**Figure 1:**
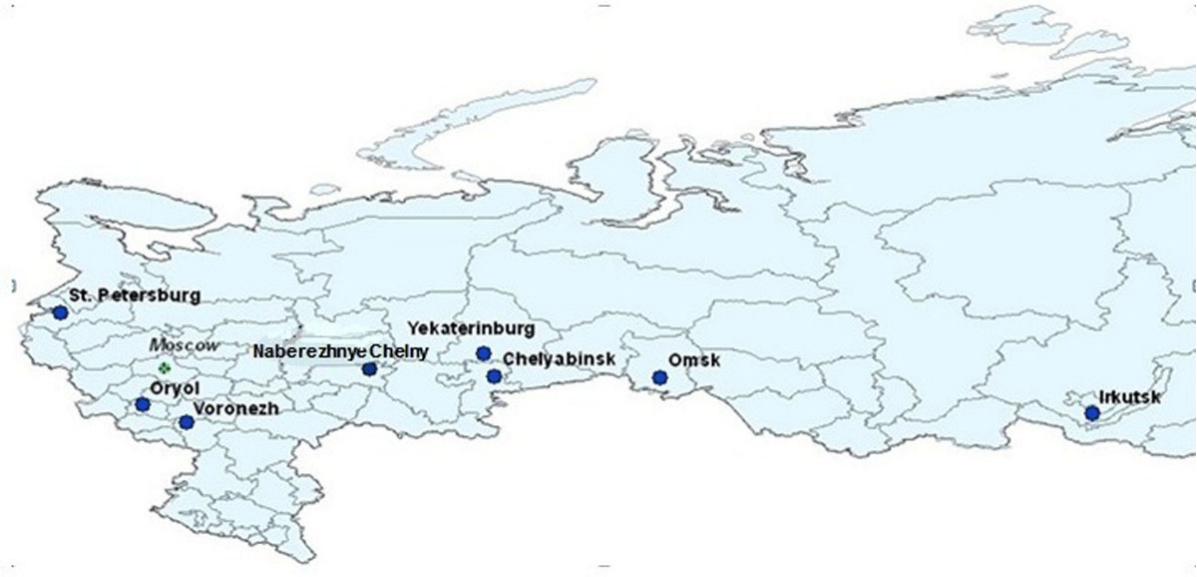


In 2008 and 2009, Russian governmental organisations and nongovernmental organisations (NGOs) conducted bio-behavioural surveillance of PWID in eight Russian cities, with funding and technical support from the World Health Organization (WHO), the Government of Finland, the United Nations Office on Drugs and Crime (UNODC), and the Russian Harm Reduction Network (ESVERO). Surveillance was carried out by administering a questionnaire and testing serosamples for HIV-1 and HCV [13]. The eight cities are geographically and demographically diverse. Four of the cities – St. Petersburg, Oryol, Voronezh, and Naberezhnye Chelny – are in European Russia and four – Yekaterinburg, Chelyabinsk, Omsk, and Irkutsk – are in Asia (Figure 1). Five have populations of more than one million inhabitants; the others have between 350,000 and 700,000 inhabitants. Observations from previous studies [14-17] and from our analysis of the distribution of HIV-1 among the PWID recruited as part of this study together suggest that individual risks, social networks, and city-to-city differences in drug markets all have influenced the epidemic situation of HIV among Russian PWID populations [13]. Observations also suggest that the same individual-, network-, and city-level factors might have influenced the spread of HCV and contributed to variations in HCV prevalence. As a result, we incorporated parameters on all three levels in the following analysis of the questionnaire data and serological findings.

## Methods

### Sampling

Bio-behavioural surveillance was conducted in eight Russian cities – St. Petersburg, Voronezh, Oryol, Naberezhnye Chelny, Yekaterinburg, Chelyabinsk, Omsk, and Irkutsk. Study participants were recruited via respondent-driven sampling (RDS), a dual-incentive chain referral sampling method that builds samples through waves of recruitment by having study participants recruit their peers. RDS relies on existing social networks. Statistical analysis of RDS samples is predicated on the assumptions that recruitment within these networks occurs at random and that more interconnected (denser) social networks will produce longer recruitment chains and more representative samples, although these assumptions may not be provable. In accordance with standard RDS methodology, “seeds” were selected for this study based on their willingness to recruit their peers into the study [18,19]. Sample sizes were set to achieve sample equilibrium and to reduce biases introduced by the non-random selection of seeds [20-22]. These were set at 300 PWID per city with the exception of St. Petersburg. There, a sample size of 400 PWID was set in recognition of the large size of the city (five million inhabitants).

People were eligible for inclusion in the study if they were aged 15 or older and reported that they had injected drugs in the last month. According to Russian regulations, anyone aged 15 or older can undergo HIV and HCV testing without a parent’s consent. Written informed consent was obtained from all study participants. To protect anonymity, participants were given the option of using an alias for the consent process. In each city, an initial group of six or seven PWID was identified through their contact with local harm reduction or HIV services. These people were asked to serve as seeds for RDS. Non-productive seeds were replaced, and in sum the sample was collected relying on 6 to 24 productive seeds in each city. Each person who agreed to participate in the study received three recruitment coupons with which to recruit his or her peers into the study. Coupon distribution ended when the sample size was reached. Participants received gifts valued at approximately 10€ for to compensate them for their time and the inconvenience of providing a blood sample. Participants also received gifts valued at €5 for each successful referral. Ethical approval was obtained from the institutional review board affiliated with St. Petersburg State University and the Federal State Institution of Science Central Research Institute of Epidemiology.

### Data collection

The data collection team in each of the eight cities consisted of one nurse, one or two interviewers, one person serving as coupon manager, and a study coordinator who supervised the other team members. Interviewers, recruited at local NGOs and AIDS centres, were social workers, psychologists, and epidemiologists who had experience working with PWID populations. In six of the cities, recruitment and data collection took place at a single site. In Irkutsk there were multiple study sites: one based at the Regional AIDS Centre and others operating from vans at fixed locations during special weekday hours. In St. Petersburg, five study sites operated at regional drug treatment clinics.

The same data collection tools were used in all eight cities. Questionnaire information was collected through face-to-face interviews. Topics included socio-demographic indicators, sexual and drug injection risk behaviours during the 30 days prior to interview, a five-item knowledge test about HIV, network size, and HIV testing history. The questionnaire was based on Family Health International guidelines for conducting behavioural surveillance [23]. It took approximately one hour to complete.

Blood specimens were drawn immediately following interviews. Each study participant received pre-test counselling, then 3-5 ml of blood were collected by venipuncture and stored in tubes marked with the participant’s unique study identification number. Blood specimens were transported to the laboratory of the Regional AIDS Centre in each participating city for testing. HCV and HIV testing were performed using enzyme-linked immunoassays; confirmatory testing was performed only for HIV. Study participants were instructed to return in 7–14 days for test results. During the post-test visit, people who received HIV-positive test results were counselled and referred to physicians working at Regional AIDS Centres for long-term care. Study participants found to be HCV antibody-positive were counselled about the meaning of the test results but no referrals for therapy were made.

### Data analysis

Data obtained directly from the questionnaire and from traditional RDS data collection from the eight cities were compiled in a single database. Individual-level frequencies, means, and medians for sociodemographic and behavioural variables of interest were calculated. Additionally, the network-level variable for self-reported network density was supplemented by a variable for each participant that recorded the length of the RDS chain that included that individual. At the city level, the mean RDS chain length and longest RDS chain in each city were calculated. Three additional dichotomous city-level variables were created in recognition of the fact that while heroin was the drug injected most often in all cities, two forms of heroin are found in Russia. The first form, which became available earlier, is created locally by extracting morphine from poppies or opium gum and converting it to heroin. Usually this form is a liquid produced in small batches [24]. The second form is commercial heroin. It originates in Afghanistan or elsewhere in Central Asia, and it is found most often as a brown or tan powder [25,26]. The two variables relating to which form of heroin predominated in each city are reciprocally related; it is yes to either one or the other. Because there is at least one report from Russia of HIV-1 incidence being associated with stimulant injection [27], we created a third variable to identify cities in which any stimulants were injected in the 30 days prior to interview by more than 10% of participants.

Our primary analytical approach was based upon the generalised linear modelling (GLM) approach employed earlier to identify factors associated with HIV prevalence in the same eight-city study population [13]. To explore the association of HCV with individual, network and city variables across the eight cities, we reviewed the statistically significant associations found between these variables and HIV. Thirteen such variables were initially selected. One variable, “knowingly injecting drugs with an HIV-positive individual“ was later removed due to a high level of non-response (> 280). We also included HIV prevalence in the model to assess the relationship between these two infections, controlling for the twelve individual-, network-, and city-level variables. Logistic regression was applied using *R* with two add-on packages, *R Commander* and *Epicalc *[28-31]. After creating the initial model with all variables, we applied backward stepwise model selection applying the *step* function in *R*. Parsimony in this algorithm is based on the Akaike Information Criteria [32], with variables removed until the lowest value is obtained. As model selection by this method must be implemented with a stable sample number, any observation with missing data in any of the variables needs to be removed.

## Results

A total of 2,596 PWID were recruited. In every city except Irkutsk, the targeted sample size was achieved. Data on sociodemographic, serological, behavioural, network-level, and city-level variables are presented in Table S1 Additional file 1. The sample was predominantly male (73%), although the proportions in the eight cities ranged from 63% to 89%. The city with the oldest participants overall was Voronezh, which had mean and median ages of 31.0 and 30, respectively; the city with the youngest participants overall was Chelyabinsk, which had mean and median ages of 24.7 and 24, respectively. More than secondary education was reported by 14% of participants and ranged from 7% to 27% across the eight cities. For the vast majority of participants, heroin was the primary drug injected, either in commercial or homemade form. Mean duration of injection was correlated with the age of the sample across the eight cities (r=0.73, p<0.05). The longest mean duration, 10.3 years, was in Voronezh; the shortest, 6.1, was in Chelyabinsk as well as in Oryol.

Overall HCV and HIV-1 serological prevalence was 71% and 30%, respectively, with substantial city-to-city variation for both viruses (Table S1 in Additional file 1). HCV prevalence ranged from 49% to 90%; HIV-1 from 3% to 64%. The association between city-level prevalence of the two viruses was analysed using the Spearman rank correlation and found to be marginally significant (r_s_=0.62, p=0.05). At the individual level, the correlation between HCV infection and HIV-1 infection was greater. Of a total of 2,569 individuals with definitive serologies for both viruses, 1,119 (44%) were positive for both and 685 (27%) were negative for both. Only 48 individuals (2%) were positive for HIV-1 but negative for HCV (data not shown).

The analysis explored four injection risk practices: injecting with a previously used syringe, sharing non-syringe injection paraphernalia, sharing water for rinsing syringes, and injecting with a syringe already loaded with dissolved drugs. It also explored two protective practices: always injecting with a sterile syringe and using locally available harm reduction services. Again, there was wide variation in the findings across the eight cities (Table S1 in Additional file 1). The most common risk was sharing non-syringe injection paraphernalia, which was practiced at least once in the 30 days prior to interview by 68% of participants. The proportion ranged from 26% in Irkutsk, which had by far the lowest proportion, to 91% in Voronezh. Among the protective practices, always injecting with a sterile syringe was reported by 69% of participants (range 61% to 86%). However, direct acquisition of sterile syringes from a harm reduction program did not appear to be a common source for syringes, since use of these services was reported by only 17% of participants (ranging from 4% in Oryol – the city with the highest rate of reported sterile syringe use – to 35% in Chelyabinsk). Perfect scores on the five-item HIV knowledge test were recorded for almost half the participants, with a range from 22% in Oryol to 65% in Chelyabinsk. Correlation between harm reduction use and HIV knowledge was highly significant at the individual level (p<0.001), but there was no correlation at the city level.

The analysis also explored several measures of PWID social networks (Table S1 in Additional file 1). The networks reported by PWID – the number of other injectors they knew by name and had encountered in the six months prior to interview – ranged in mean size across the eight cities from 11 in Chelyabinsk to 50 in Yekaterinburg. This self-report of network density was not correlated with either mean recruitment chain length (r_s_=-0.29, p>0.05) or with the length of the longest chain in that city (r_s_=0.14, p>0.05). At the individual level we compared self-reported network density with the length of that individual’s recruitment chain using linear regression and found that there was a negative association between the two variables (β = -0.042±0.017, p=0.015).

At the city level, commercial heroin was predominant in all but two cities – Oryol and Voronezh. As the data presented in Table S1 Additional file 1 demonstrate, any use of the non-predominant form in the 30 days prior to interview by more than 10% of participants was found in only two cities. In Yekaterinburg, although commercial heroin was predominant, 20% of participants reported injecting homemade heroin in the 30 days prior to interview. In Oryol, although homemade heroin was predominant, 31% of participants reported injecting commercial heroin in the 30 days prior to interview. In three cities – Yekaterinburg, Naberezhnye Chelny, and Oryol – more than 10% of participants reported stimulant injection in the 30 days prior to interview.

Taking HCV prevalence as the dependent variable, we created a multivariate model that included the demographic variables and the individual-, network-, and city-level variables that were significantly associated with HIV-1 prevalence in bivariate analysis identified in our earlier work [13]. Due to modelling requirements, data from 2,457 participants were included in the final model. The most parsimonious model, presented in Table 1, included eight variables. Those most strongly associated with HCV prevalence were HIV-1 prevalence and a longer duration of injection drug use. Risk practices significantly associated with HCV prevalence were sharing non-syringe injection paraphernalia and rinse water. Injecting with a used syringe was associated with *not* being infected. At the network level, having a larger social network was associated with HCV prevalence, but being part of a longer recruitment chain was inversely associated with HCV prevalence. At the city level, the existence of a higher proportion of stimulant injectors was also inversely associated with HCV prevalence.

**Table 1:** Multivariate modeling of individual-, network-, and city-level variables associated with HCV seropositive status among PWID in eight Russian cities (N=2,457)

	Estimate	Standard Error	z value	Pr(>|z|)
(Intercept)	0.366	0.200	1.825	0.07

Positive HIV-1 serology	2.131	0.166	12.803	<0.0001

Years Injecting	0.038	0.010	4.047	<0.0001

Injected with used syringe	-0.383	0.159	-2.408	0.016

Shared non-syringe injection paraphernalia	0.333	0.107	3.127	0.002

Shared water for rinsing syringes	0.495	0.158	3.133	0.002

Social network size	0.005	0.002	2.717	0.007

Mean recruitment chain length	-0.003	0.002	-2.222	0.03

Stimulant use >10%	-0.402	0.110	-3.644	<0.001

Akaike Information Criterion=2,565

## Discussion

The data demonstrate that HCV is entrenched in populations of PWID in Russia and is endemic in some locations. In the final multivariate model, eight factors were associated with HCV prevalence: co-infection with HIV-1, longer duration of injection drug use, sharing non-syringe injection paraphernalia, sharing rinse water, injecting with new syringes, larger individual social network size, smaller city-level mean chain length, and lower injection of stimulants at the city level. The factors most closely associated with HCV infection are duration of injecting drug use and HIV-1 co-infection.

We started with the hypothesis that HCV prevalence levels and factors associated with higher prevalence would be similar to our previously published findings for HIV-1 [13]. Our analysis showed that HCV seroprevalence was associated with HIV-1 seroprevalence at both the individual and city level. The correlation was greater at the individual than at the city level. The strong individual-level correlation of HCV- and HIV-positive serostatus and the higher prevalence of HCV are consistent with data from many parts of world that demonstrate that HCV is spread more quickly than HIV-1 by unsafe injection and that HCV prevalence increases among PWID in advance of rising HIV-1 prevalence [33-36]. This is not surprising given that HCV is considered more infectious than HIV-1 by a factor of ten [37]. This difference may be the key feature in explaining the higher prevalence of HCV given that differences in HCV and HIV-1 survival in syringes are minor [38-41].

The longer a person has injected, the more likely he or she is to have been exposed to HCV via unsafe injection practices, which were quite common among study participants. These same practices can result in HIV-1 transmission. Studies that have explored HCV prevalence and incidence as a function of time since initiation of injection have found incidence rates in excess of 20% per year beginning in the first few years after initiation [33,35,42-44]. Although no comparable study has been conducted among Russian PWID, the international nature of the citations suggests that a similar pattern among Russian PWID is quite likely.

However, one of the key factors associated with HIV-1 prevalence, transition at the city level to commercial heroin from homemade heroin, was not associated with HCV prevalence. Further research at the virological and social levels will be needed to determine why the pattern is not the same for the two drugs although both are transmitted by unsafe injection.

HCV prevalence was inversely associated with the city-level proportion of PWID injecting stimulants. This may be the result of the type of stimulants injected. Almost all stimulants are of the amphetamine type (ATS) derived from ephedrine, pseudoephedrine, or phenylephrine; cocaine access and injection are rarely reported. Two features of ATS, especially methamphetamine, can reduce the transmission probability for HCV. First, methamphetamine has a longer biological half-life than heroin and therefore may be injected less often (in contrast to cocaine with a shorter half-life and higher injection frequency than seen for heroin). Second, the home manufacture ATS, which is the most common source for ATS in Russia, is associated with an acidic product that has been proven virucidal for HIV-1 [45]. This situation might also reduce HCV transmissibility.

In our study, the risk practices themselves were less strongly associated with HCV infection, but the timeframe for which study participants were asked about injection practices was the past 30 days, whereas the HCV infection itself might have occurred at any time since the initiation of drug injection. An unexpected finding of our study was that not sharing syringes is associated with HCV prevalence. A possible explanation for this reverse correlation may relate to the eight-year mean duration of injecting drug use in the study population. People who inject drugs for longer periods may have had greater exposure to safe injection messages that focus primarily on not sharing syringes and on using sterile syringes for every injection rather than calling attention to other pathways for disease transmission such as sharing rinse water. It may also be the case that once individuals learn that they have HCV or HIV-1, they act altruistically to reduce the risk of transmitting the virus to others. This issue could be further explored through qualitative research that examines PWID attitudes and beliefs about safer injection practices.

The study has a number of limitations. The reliance on self-reporting of injection risk and protective practices is clearly subject to recall and social desirability biases. We found evidence for this in some instances. The data analysis utilised two mutually exclusive categories that allow a logic check: an individual cannot have always used sterile syringes and concurrently have injected with a used syringe or with a pre-filled syringe in the past 30 days. Therefore, the combined percentages for these two combinations of variables should not exceed 100%. We found this rule violated in 3 of 16 possible cases. The percentage of people reporting using only sterile syringes and using a prefilled syringe exceeded 100% in Yekaterinburg (107%) and Oryol (123%); the same was the case for using only sterile syringes and injecting with a used syringe in St. Petersburg (109%). However, the internal logic check did not identify over-reporting of sterile syringe use or under-reporting of risky practices in 13 of 16 test cases, so misreporting injection behaviours may be only a minor problem. A second limitation is the use of RDS to determine the study population. This method relies on social connections among PWID, and consequently there is a loss of independence in the data because socially mediated factors such as injection practices and HCV and HIV-1, which are socially transmitted viruses, tend to be similar among members of the same social network [46-48]. Since we do not know the true network structure of PWID, we cannot calculate the impact the loss of independence has on the data and therefore cannot claim that the findings are representative of PWID populations in the eight cities or of Russian PWID as a whole. In addition, self-reported network size was not associated with recruitment success, suggesting that features of RDS unrelated to the number of known potential contacts had a greater influence than network size on recruitment. This calls into question the validity of applying statistical tests to adjust estimates based on network size. The assumption that recruitment occurred at random within the recruiters’ social network also cannot be proven given the nature of the data that were collected. A third limitation arises from the use of GLM for model selection, as this method requires a stable sample number. This resulted in an approximately 5% reduction in sample size to produce the most parsimonious model. However, we believe that the reduction made no difference for the identification of those variables significantly associated with HCV prevalence, since there was no consistent pattern to the data loss.

Despite its limitations, the study provides an important contribution to understanding the penetrance of HCV into PWID populations in eight Russian cities. The prevalence range, from 49% to 90%, is at the high end of the range found across Europe in studies at both the national and subnational levels [49]. Factors associated with being HCV seropositive are similar to those reported elsewhere in Europe [50]. Taken together, the data suggest that although the epidemic of injection drug use arrived later in Russia than in Western Europe, the absence of widespread early government-supported prevention efforts has created conditions for substantial HCV transmission. More work is needed to turn these findings into usable prevention messages and activities, but it is at least obvious that reducing transmission of HCV will require improving access to harm reduction programs, teaching and encouraging better injection hygiene practices, making clean syringes more widely available, and expanding access to care.

Each of the steps needed to ameliorate the health impacts of the HCV epidemic among PWID will require substantial effort in the Russian context. Harm reduction programs in Russia have historically been conducted by NGOs with funding from sources external to Russia. At the federal level, there is little support for harm reduction approaches [51,52]. Although there are a few examples of programmatic success when local governments have supported NGO-operated programs in Kazan and St. Petersburg [53,54], this model has not been widely adopted. Our finding of a strong correlation between attendance at harm reduction programs and HIV knowledge suggests that programs should be encouraged to promote HCV prevention knowledge, especially regarding HCV-specific syringe hygiene issues. For example, the prolonged infectivity of HCV on surfaces [55] requires that clean surfaces be maintained when individuals prepare drugs for injection in places where other people also inject. For the same reason, post-injection hygiene is necessary to reduce the presence of HCV on surfaces. We have shown that an intervention that provides proper materials to stanch the flow of blood following injections can be implemented at harm reduction programs and accepted by their customers who inject drugs [56] .Finally, emphasis should be placed on the process of injecting drug, not just the items involved because when drugs are prepared using a contaminated syringe and shared with others, it does not protect the others if they have sterile syringes – the drug solution has become contaminated [57,58].

It is possible, however, that expanding attempts to publicly promote injection hygiene and teach safer injection might cause programs to run afoul of the Russian federal law that makes it illegal to promote drug use [59]. Another consideration is that while syringes can be purchased without a prescription in Russia, this is often prevented in practice by pharmacists’ reluctance to sell to people they think might be PWID and by police interference with syringe purchases [51,60,61]. It might be possible to work with pharmacists’ associations to encourage them to serve as public health actors and thereby overcome their reluctance to provide syringes more readily [62]. Our preliminary studies in St. Petersburg suggest that this is possible [60].

Lastly, increasing access to treatment with the new generation of HCV antiviral medications has the potential to cure more cases of HCV infection and reduce transmission on a larger scale. Russian law allows for free treatment of some groups of Russian citizens, but none of the new medications, although available in Russia, are yet on the approved list [63]. When the newer medications do become available, they will be very costly, and the tremendous stigma directed against PWID will need to be overcome in making a compelling case for why limited health system resources should be expended on PWID.

## Conclusions

The HCV epidemic among PWID in Russia is already quite advanced. While increasing access to harm reduction service to reduce the risk for further transmission may have some impact on the epidemic, true control will occur only through a multifaceted approach that includes treatment to eliminate virus from those already infected, expanded syringe access through pharmacies and syringe exchange programs, and effective treatment for opioid addiction. The likelihood of these occurring in Russia in the near future is small given the historic resistance of Russian health authorities to proven methods for control of viral transmission among PWID that are generally accepted in most of the rest of the world.

## Authors’ contributions

RH designed the analysis and was primarily responsible for writing the manuscript. KE supervised data collection, compiled the database, and conducted some of the statistical analyses, RB assisted with data analysis and modelling the relationship between HCV and other factors, OSL played a major role in organizing the data collection and participated in analytical decisions and drafting of the manuscript.

## Competing interests

The authors declare that they have no competing interests.

## Supplementary Material

Additional File 1 Table S1. Microsoft Word Document. Click here for file

## Declarations

This article has been published as part of *BMC Infectious Diseases* Volume 14 Supplement 6, 2014: Viral Hepatitis in Europe. The full contents of the supplement are available online at http://www.biomedcentral.com/bmcinfectdis/supplements/14/S6. The publication charges for this supplement were funded by AbbVie as an unrestricted grant to Rigshospitalet, the University of Copenhagen. AbbVie further funded the printing of the supplement with additional financial support from the Drug Prevention and Information Programme (DPIP) of the European Union.
