# Supplementary material for: Hepatitis C virus seroprevalence among people who inject drugs and factors associated with infection in eight Russian cities
Source: BMC Infect Dis. 2014 Sep 19;14(Suppl 6):S12. doi: 10.1186/1471-2334-14-S6-S12 (PMC4178532; doi:10.1186/1471-2334-14-S6-S12)
Supplement: Additional File 1 — Table S1. Microsoft Word Document. [file 1471-2334-14-S6-S12-S1.doc]

**TABLE S1. Serological findings and demographic and behavioural characteristics of study participants in eight Russian cities**

|  | Irkutsk | Omsk | Chelyabinsk | Yekaterinburg | Naberezhyne Chelny | Voronezh | Oryol | St. Petersburg | Total |
| --- | --- | --- | --- | --- | --- | --- | --- | --- | --- |
| Sample size | 279 | 350 | 306 | 300 | 341 | 309 | 300 | 411 | 2,596 |
| INDIVIDUAL-LEVEL VARIABLES | | | | | | | | |  |
| Male sex | 193 (69%) | 262 (75%) | 272 (89%) | 189 (63%) | 259 (76%) | 222 (72%) | 210 (70%) | 300 (73%) | 1,907 (73.4%) |
| Age  Mean  Median | 26.7  26 | 29.7  29 | 24.7  24 | 28.1  28 | 28.0  28 | 31.0  30 | 26.0  26 | 29.9  29 | 28.1  28 |
| Education beyond secondary | 38 (14%) | 25 (7%) | 34 (11%) | 30 (10%) | 72 (21%) | 45 (15%) | 81 (27%) | 29 (7%) | 354 (13.6%) |
| Duration of injection drug use: mean years | 9.1 | 9.2 | 6.1 | 8.4 | 7.2 | 10.3 | 6.1 | 9.4 | 8.0 |
| SYRINGE-BORNE VIRUS PREVALENCE | | | | | | | | |  |
| Positive HCV serology | 236 (85%) | 252 (72%) | 152 (51%) | 270 (90%) | 164 (49%) | 222 (72%) | 183 (61%) | 358 (90%) | 1,837 (70.8%) |
| Positive HIV-1 serology | 159 (57%) | 30 (9%) | 46 (15%) | 193 (64%) | 42 (13%) | 8 (3%) | 44 (15%) | 244 (59%) | 766 (29.5%) |
| INJECTION RISK BEHAVIOURS AND PROTECTIVE BEHAVIOURS, PAST 30 DAYS | | | | | | | | |  |
| Injected with used syringe | 64 (23%) | 116 (33%) | 91 (29%) | 40 (13%) | 79 (23%) | 96 (31%) | 31 (10%) | 179 (41%) | 696 (26.8%) |
| Shared non-syringe injection paraphernalia | 73 (26%) | 214 (61%) | 184 (60%) | 195 (65%) | 273 (80%) | 281 (91%) | 227 (76%) | 316 (77%) | 1,763 (67.9%) |
| Shared water for rinsing syringes | 89 (32%) | 154 (44%) | 116 (38%) | 114 (38%) | 106 (31%) | 111 (36%) | 36 (12%) | 255 (62%) | 981 (37.8%) |
| Injected with prefilled syringe | 75 (27%) | 120 (34%) | 83 (27%) | 82 (27%) | 85 (25%) | 178 (58%) | 111 (37%) | 86 (21%) | 820 (31.6%) |
| Injected stimulants[[1]](#endnote-2) | 0 | 14 (4%) | 30 (10%) | 89 (30%) | 62 (18%) | 7 (2%) | 79 (26%) | 30 (7%) | 311 (12.0%) |
| Always used sterile syringes | 177 (63%) | 215 (61%) | 192 (63%) | 241 (80%) | 229 (67%) | 193 (62%) | 257 (86%) | 280 (68%) | 1,784 (68.7%) |
| Any use of harm reduction services | 33 (12%) | 55 (16%) | 107 (35%) | 76 (26%) | 62 (18%) | 38 (12%) | 14 (4%) | 50 (12%) | 435 (16.8%) |
| Drug treatment  Ever  Last year | 162 (58%)  126 (45%) | 60 (17%)  26 (7%) | 89 (29%)  49 (16%) | 45 (15%)  24 (8%) | 204 (60%)  146 (43%) | 105 (34%)  93 (30%) | 63 (21%)  27 (9%) | 152 (37%)  111 (27%) | 880 (33.9%)  602 (23.2%) |
| Perfect HIV knowledge (five-item test) | 145 (52%) | 123 (35%) | 199 (65%) | 108 (36%) | 204 (60%) | 198 (64%) | 66 (22%) | 239 (53%) | 1,282 (49.4%) |
| NETWORK-LEVEL VARIABLES | | | | | | | | |  |
| Mean social network size, past 6 months | 35.8 | 42.4 | 10.9 | 49.7 | 15.7 | 21.9 | 19.0 | 17.2 | 26.1 |
| Mean recruitment chain length | 33.0 | 100.8 | 111.9 | 55.8 | 60.7 | 83.9 | 99.1 | 39.9 | 72.5 |
| Longest chain (% of sample) | 169 (61%) | 96 (27%) | 65 (20%) | 90 (30%) | 164 (48%) | 156 (50%) | 85 (28%) | 120 (29%) |  |
| CITY-LEVEL VARIABLES | | | | | | | | |  |
| Commercial heroin dominant  (% of PWID reporting any use, past 30 days) | Yes (98%) | Yes (99%) | Yes (98%) | Yes (99%) | Yes (100%) | No (1%) | No (31%) | Yes (95%) |  |
| Homemade heroin dominant  (% of PWID reporting any use, past 30 days) | No (7%) | No (7%) | No (7%) | No (20%) | No (3%) | Yes (97%) | Yes (79%) | No (<1%) |  |
| Stimulant injection >10% | No | No | No | Yes | Yes | No | Yes | No |  |

1. This includes methamphetamine, methcathinone, cathinone, and cocaine [↑](#endnote-ref-2)
